# Living Within the Macrophage: Dimorphic Fungal Pathogen Intracellular Metabolism

**DOI:** 10.3389/fcimb.2020.592259

**Published:** 2020-10-16

**Authors:** Qian Shen, Chad A. Rappleye

**Affiliations:** ^1^Department of Biology, Rhodes College, Memphis, TN, United States; ^2^Department of Microbiology, Ohio State University, Columbus, OH, United States

**Keywords:** *Histoplasma*, *Paracoccidioides*, macrophage, phagosome, metabolism, fungal pathogen, gluconeogenesis, nutritional immunity

## Abstract

*Histoplasma* and *Paracoccidioides* are related thermally dimorphic fungal pathogens that cause deadly mycoses (i.e., histoplasmosis and paracoccidioidomycosis, respectively) primarily in North, Central, and South America. Mammalian infection results from inhalation of conidia and their subsequent conversion into pathogenic yeasts. Macrophages in the lung are the first line of defense, but are generally unable to clear these fungi. Instead, *Histoplasma* and *Paracoccidioides* yeasts survive and proliferate within the phagosomal compartment of host macrophages. Growth within macrophages requires strategies for acquisition of sufficient nutrients (e.g., carbon, nitrogen, and essential trace elements and co-factors) from the nutrient-depleted phagosomal environment. We review the transcriptomic and recent functional genetic studies that are defining how these intracellular fungal pathogens tune their metabolism to the resources available in the macrophage phagosome. In addition, recent studies have shown that the nutritional state of the macrophage phagosome is not static, but changes upon activation of adaptive immune responses. Understanding the metabolic requirements of these dimorphic pathogens as they thrive within host cells can provide novel targets for therapeutic intervention.

## Introduction

*Histoplasma* and *Paracoccidioides* species cause respiratory and systemic disease in mammals (histoplasmosis and paracoccidioidomycosis, respectively). Disease results from inhalation of aerosolized fungal conidia from the environmental mold form. Severity of disease ranges from subclinical to acute, and is largely a function of the dose inhaled and the immunological defenses of the host (Kauffman, 2009; Queiroz-Telles and Escuissato, 2011; Salzer et al., 2018). In immunocompromised individuals (e.g., HIV patients), infection can result in life-threatening disseminated disease. Recent phylogenetic analyses based on genome sequencing efforts have split the original *Histoplasma capsulatum* and *Paracoccidiodies brasiliensis* designations into additional species (Van Dyke et al., 2019). Although some physical and biochemical differences exist among these species groups, in this review we will refer to studies in each fungus by the respective genus name unless necessary to specifically highlight findings unique to a species.

The *Histoplasma* and *Paracoccidiodies* genera comprise closely related fungi which are characterized by thermal dimorphism. Conidia, produced by the environmental mycelial forms, convert into pathogenic yeast forms in response to the elevated temperature following inhalation by a mammalian host. The yeasts are budding yeasts, with *Paracoccidioides* yeasts typically characterized by multiple budding daughter cells around a central yeast body. A hallmark of these dimorphic fungal pathogens is the link between pathogenesis and conversion into yeasts (Medoff et al., 1987; Maresca and Kobayashi, 1989; Aristizabal et al., 1998; Borges-Walmsley et al., 2002).

During infection, *Histoplasma* and *Paracoccidioides* yeast cells are phagocytosed by cells of the innate immune system. Studies with cultured phagocytes (i.e., macrophages) show roughly 10%-30% of *Paracoccidioides* yeasts are taken up by macrophages (Soares et al., 2010; da Silva et al., 2016) while up to 60% of *Histoplasma* yeasts are phagocytosed (Newman et al., 1990; Garfoot et al., 2016) within 60–90 min in tissue culture conditions. Studies of respiratory infection in mice show that nearly all *Histoplasma* yeasts are found within CD45^+^ phagocytes (Deepe et al., 2008). Thus, *Histoplasma* yeasts reside almost exclusively within phagocytes during infection *in vivo*, and many, but not all *Paracoccidioides* yeasts are intracellular. Non-activated host phagocytes are relatively ineffective in killing and controlling *Histoplasma* and *Paracoccidioides* yeasts. Activation of phagocytes during adaptive immunity by T helper cell type 1 (Th1) is typically required for host control of these fungi (Allendoerfer and Deepe, 1997, 1998; Souto et al., 2000; Calich et al., 2008; de Castro et al., 2013) thereby distinguishing *Histoplasma* and *Paracoccidioides* from opportunistic fungal pathogens, which are readily controlled without T-cell activation. Recently, IL-17 signaling was shown to also contribute to the control of *Paracoccidioides* (Tristão et al., 2017).

To effectively use phagocytes as host cells, *Histoplasma* and *Paracoccidioides* yeasts express mechanisms that facilitate binding to host phagocytes and survival of macrophage defenses. Although the fungal cell wall contains β-glucans which can alert the immune system to the presence of a fungal invader, *Histoplasma* yeasts effectively hide this β-glucan signature by overlaying the immunostimulatory β-glucans with α-glucan polysaccharides (Rappleye et al., 2004, 2007). In addition, *Histoplasma* yeasts secrete an endoglucanase (Eng1) that trims away remaining exposed β-glucans (Garfoot et al., 2016). Although a similar mechanism has not been shown for *Paracoccidioides*, the cell wall of *Paracoccidioides* yeasts also has a high content of α-glucan (Kanetsuna et al., 1972) and decreased α-glucan is associated with decreased virulence (San-Blas et al., 1977). *Histoplasma* expresses two extracellular antioxidant enzymes, Sod3 and CatB, which together effectively eliminate phagocyte-derived reactive oxygen (Youseff et al., 2012; Holbrook et al., 2013) and facilitate *Histoplasma* survival during uptake by macrophages. Consistent with the pathogenesis-enabling role of these mechanisms, α-glucan production and secretion of Eng1, Sod3, and CatB characterize the virulent yeasts, but not the avirulent mycelial cells.

Pathogenic yeast not only survive the encounter with phagocytes, but also use the phagocyte as a permissive niche during infection. As long-term intracellular residents of the macrophage, *Histoplasma* and *Paracoccidioides* yeasts must adapt their metabolism to nutrients available in the phagosome. The phagosome environment is generally assumed to be nutrient poor, yet these fungi are able to scavenge sufficient carbon, nitrogen, sulfur, and micronutrients from the host cell to support fungal growth and replication. Many studies have used gene expression profiling to infer which metabolic pathways are active during residence within the phagosome. Interpretation of differentially expressed genes, however, is complicated by the *in vitro* growth condition used as the comparison. Furthermore, a large number of genes that characterize the pathogenic yeast phase of the dimorphic fungi are controlled by phase-differentiation factors such as the Ryp proteins (Shen and Rappleye, 2017; Beyhan and Sil, 2019) suggesting that some metabolic genes may not be regulated by the nutritional environment, but instead their expression is set by the differentiation state (i.e., yeasts vs. mycelia). Genetic studies, and the functional tests it facilitates, are a means to more conclusively determine the metabolism used by intracellular yeasts to parasitize the macrophage. Recent studies highlighted below integrate key findings that define the metabolic pathways required for intracellular yeast proliferation, and by extension, the host substrates that are consumed by these fungal pathogens during infection.

## Intraphagosomal Carbon Metabolism

### Glycolysis

Most *in vitro* fungal growth media is based on glucose as the primary carbon source since glucose affords rapid fungal growth. Although this rich carbon source may reflect some host environments (e.g., the bloodstream), other host niches, including the macrophage phagosome are characterized by alternative carbon sources. Gene expression studies in which specific metabolic pathways are up-regulated in specific host environments can reveal the presence of different carbon sources. Bailão et al. (2006) conducted the first study with *Paracoccidioides* yeasts to determine which genes are up-regulated during infection. Comparing the gene expression profile of yeasts grown in human blood to yeasts grown *in vitro*, Bailão et al. (2006) found that glyceraldehyde 3-phosphate dehydrogenase (GAPDH) was up-regulated in blood. Since GAPDH is involved in both glycolysis and gluconeogenesis, it remains unclear if glucose catabolism contributes to *Paracoccidioides* infection of host cells. While some *Paracoccidioides* cells may be taken up by phagocytes in blood (e.g., neutrophils), significant numbers may remain extracellular. Thus, the results might reflect the transcriptional responses of a mixed population consisting of both extracellular yeasts and intracellular yeasts from different types of phagocytes. Thus, the high concentration of extracellular glucose in the blood, not the intracellular phagosome environment, may be the primary driver of GAPDH up-regulation.

Profiling of yeasts resident within macrophages offers a more direct assessment of the transcriptional profiles supporting intraphagosomal growth. Tavares et al. (2007) conducted a differential expression study comparing the gene expression profile of *Paracoccidioides* yeasts grown in murine peritoneal macrophages and those grown *in vitro* in rich media. Using a transcriptional microarray, the authors found that the gene encoding the glycolysis specific enzyme phosphofructokinase (*PFK1*) was down-regulated in intracellular *Paracoccidioides* yeasts. A separate transcriptional study that profiled *Paracoccidioides* yeasts from the lung identified a sugar transporter that was down-regulated relative to yeasts grown *in vitro* in a glucose-rich medium [2% glucose (Lacerda Pigosso et al., 2017)]. This suggests that glucose catabolism is not a primary metabolic pathway for *Paracoccidioides* intracellular growth. However, in the Tavares study (Tavares et al., 2007), RT-PCR data was unable to confirm the microarray results. Proteomic analysis of *Paracoccidioides* intracellular yeasts found that hexokinase (Hxk1) was down-regulated compared to yeast grown in a glucose-rich medium *in vitro* consistent with decreased metabolism of sugars (Parente-Rocha et al., 2015). The transcriptional signature of intracellular *Histoplasma* yeasts also shows down-regulation of a glycolytic response when growing within macrophages (Shen et al., 2020). As with *Paracoccidioides, PFK1* was down-regulated in *Histoplasma* yeasts. Genes encoding other glucose-catabolism factors including pyruvate kinase (*PYK1*) and the hexose/glucose kinases (*HXK1* and *GLK1)* were also down-regulated. The finding that multiple components of glycolysis showed decreased expression provides stronger support for the metabolism of alternative carbon sources during intracellular proliferation. As an independent indicator of the lack of hexose catabolism by intracellular *Histoplasma* yeasts, metabolic assays (i.e., Seahorse assays) of *Histoplasma* yeasts isolated from macrophages showed the yeasts were glycolytically inactive (Shen et al., 2020) consistent with the transcriptional profiles. Definitive evidence of the lack of hexose catabolism by intracellular yeasts came from the analysis of glycolysis-deficient *Histoplasma* yeasts. Simultaneous depletion of *Histoplasma*'s only hexose/glucose kinases (Hxk1 and Glk1) impaired the ability of *Histoplasma* yeast to grow on glucose as the carbon source *in vitro*, but Hxk1 and Glk1 loss did not prevent *Histoplasma* yeast growth within macrophages (Shen et al., 2020). Most revealing, the depletion of Hxk1 and Glk1 and no effect on *Histoplasma* respiratory infection *in vivo* (Shen et al., 2020). These data demonstrate that *Histoplasma* yeasts, and likely also *Paracoccidioides* yeasts, do not catabolize hexoses to grow within macrophages. As both *Histoplasma* and *Paracoccidioides* efficiently use glucose *in vitro*, this suggests that it is unlikely that hexoses are available to yeasts within the macrophage phagosome.

### Gluconeogenesis

The lack of hexose utilization suggests that gluconeogenic substrates are likely metabolized by intracellular yeasts to provide energy and essential biomolecules including glucans for the polysaccharide-rich cell wall. A combined transcriptomic and proteomics study compared *Paracoccidioides* yeasts isolated from mouse lungs after 6 h of infection to yeasts grown in a glucose- and peptide-rich medium *in vitro* [Brain Heart Infusion medium (Lacerda Pigosso et al., 2017)]. The *PCK1* gene, encoding phosphoenolpyruvate carboxykinase which catalyzes the first committed step of gluconeogenesis, was transcriptionally up-regulated *in vivo*, suggesting the yeasts resided in a glucose-depleted environment. However, the proteomics analysis failed to detect Pck1 at the protein level in yeasts isolated from the lung. As only a portion of *Paracoccidioides* yeasts get internalized by phagocytes, it is unclear whether the up-regulation of *PCK1* reflects the intracellular or extracellular nutritional environment during *in vivo* infection and whether 6 h of infection is sufficient for transcriptional or proteomic adaptive responses. One of the limitations of this study was the recovery of low numbers of yeasts which could impact the sensitivity of their analyses. Many normally-abundant proteins were not detected in the proteomic data and key enzymes involved in the central carbon metabolism (i.e., pyruvate kinase, citrate synthase, and isocitrate dehydrogenase) that should be abundant in yeast cells, regardless of growth condition, were not recovered from the proteome data. No conclusions can be made from the absence of data, but an independent study of intramacrophage *Paracoccidioides* yeasts also showed up-regulation of the *PCK1* gene after 9 h of infection (Derengowski et al., 2008). In addition, Parente-Rocha et al. (2015) found that gluconeogenesis-specific enzyme fructose-1,6-bisphosphatase was modestly increased (1.5-fold) in *Paracoccidioides* yeasts recovered from macrophages, providing further support of gluconeogenesis-based metabolism in intracellular yeasts.

**Figure 1:**
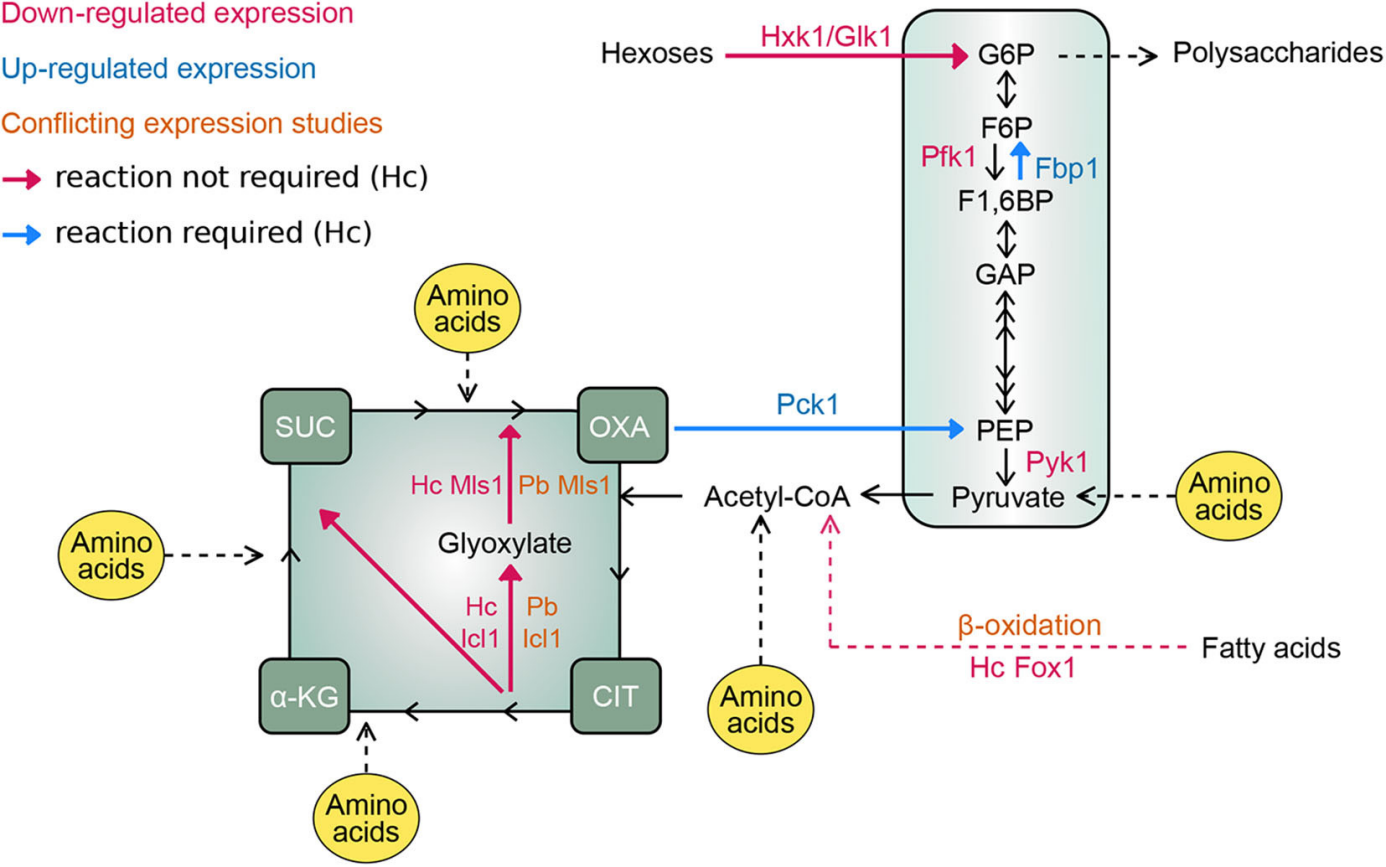
Intramacrophage carbon metabolism of *Histoplasma* and *Paracoccidioides*. Schematic map depicts the central metabolic pathways for the metabolism of different carbon-containing molecules with key carbon intermediates shown. Enzymes catalyzing critical steps of glycolysis (Hxk1/Glk1, Pfk1, and Pyk1), gluconeogenesis (Pck1 and Fbp1), and fatty acid utilization (Fox1 for β-oxidation of fatty acids, and Icl1 and Mls1 for the glyoxylate cycle for incorporation of acetyl-CoA into biomolecules) are indicated. Names of enzymes are colored based on the down-regulation (magenta text) or up-regulation (blue text) in fungal cells within macrophages. *in situ*ations where expression data is conflicting, text is colored orange. For expression data that is discrepant between *Paracoccidioides* and *Histoplasma*, the respective genes are provided by a species prefix (Pb and Hc, respectively). Arrows indicate the flow of carbon through metabolic pathways. Enzymatic reactions that are not required for intracellular growth are indicated with magenta arrows. Reactions necessary for proliferation in the phagosome and for infection *in vivo* are indicated with blue arrows. Points of entry for amino acids as potential carbon substrates are indicated in yellow. Enzyme abbreviations: Hxk1, hexokinase; Glk1, glucokinase; Pfk1, phosphofructokinase; Pyk1, pyruvate kinase; Pck1, phosphoenolpyruvate carboxykinase; Fbp1, fructose 1,6-bisphosphatase; Fox1, fatty acyl-CoA oxidase; Icl1, isocitrate lyase. Metabolite abbreviations: G6P, glucose 6-phosphate; F6P, fructose 6-phosphate; F1, 6BP, fructose 1,6-bisphosphate; GAP, glyceraldehyde 3-phosphate; PEP, phosphoenolpyruvate; CIT, citrate; α-KG, α-ketoglutarate; SUC, succinate; OXA, oxaloacetate.

Studies of *Histoplasma* yeasts isolated from macrophages 24 h after infection shows *PCK1* expression is up-regulated in intracellular yeasts (Shen et al., 2020). Elimination of Pck1 activity prevents growth of *Histoplasma* yeasts within macrophages but not *in vitro* growth in glucose-containing medium confirming that the phagosome environment lacks hexose substrates (Shen et al., 2020). *Histoplasma* yeasts lacking Pck1 function are severely attenuated in a murine model of respiratory infection as early as 2 days post-infection and this growth defect *in vivo* persisted throughout the entire 8-day infection period, indicating that the *Histoplasma*-containing phagosome remains depleted of hexose substrates. The gluconeogenesis-specific fructose-1,6-bisphosphatase (Fbp1) is also required for *Histoplasma* yeast virulence in macrophages and in mice (Shen et al., 2020). These findings are consistent with the results that glycolytic enzymes are not required for *Histoplasma* full virulence *in vivo*. Collectively, these findings indicate that *Histoplasma* yeasts rely on metabolizing gluconeogenic carbon sources to proliferate within macrophages (Figure 1) and this metabolism likely characterizes intracellular *Paracoccidioides* yeasts.

### Fatty Acids Utilization

What are the gluconeogenic substrates derived from the host that support yeast proliferation within phagosomes? One potential source is host fatty acids. These long-chain carbon molecules can be metabolized to acetyl-CoA (β-oxidation) which subsequently enters the TCA cycle to generate energy or can be assimilated into carbon biomass through the glyoxylate shunt. The up-regulation of the glyoxylate shunt can indicate utilization of fatty acids as the carbon source. In one study (Derengowski et al., 2008), the expression of key genes involved in glyoxylate shunt [isocitrate lyase (*ICL1*) and malate synthase (*MLS1*)] in *Paracoccidioides* yeasts was measured during infection of J774 macrophages using semi-quantitative RT-PCR. Expression of *ICL1* increased ~3-fold in macrophages compared to growth in Fava-Neto medium (a rich medium containing high glucose, peptides, and amino acids). However, expression of *MLS1* showed only a minor increase in expression. Isocitrate lyase protein (Icl1) increased 1.6-fold in *Paracoccidioides* yeasts recovered from J774 macrophages (Parente-Rocha et al., 2015). The up-regulation of *ICL1* is consistent with results profiling *Candida albicans* and *Cryptococcus neoformans* yeasts when interacting with macrophages (Lorenz et al., 2004; Fan et al., 2005). However, Tavares et al. (2007) reported conflicting results that *ICL1* was not up-regulated in *Paracoccidioides* yeasts within peritoneal macrophages, possibly due to *in vitro* growth conditions used for comparison. Expression profiling of *Paracoccidioides* yeasts 6 h after infection of mice also found an indication of the up-regulation of the glyoxylate shunt, but only the *MLS1* gene, not *ICL1* (Lacerda Pigosso et al., 2017). Growth of *Paracoccidioides* yeasts on acetate *in vitro* increases both *ICL1* and *MSL1* expression by a similar magnitude (Derengowski et al., 2008), suggesting the one-sided increases in *ICL1* or *MLS1* expression by *Paracoccidioides* yeasts in macrophages or *in vivo* do not indicate simple induction of the glyoxylate cycle. These inconsistencies complicate inferences about the role of the glyoxylate cycle in intracellular *Paracoccidioides* yeasts.

As with the glyoxylate cycle, indications of fatty acid catabolism by *Paracoccidioides* yeasts are also variable. Enzymes catalyzing the breakdown of fatty acids via β-oxidation have been examined for *Paracoccidioides* yeasts both in macrophages and *in vivo*. While enoyl-CoA hydratase (which catalyzes the second step in fatty acid metabolism) was up-regulated in *Paracoccidioides* yeasts from J774 macrophages, 3-keyoacyl-CoA thiolase (which catalyzes the last step liberating acetyl-CoA) was down-regulated in these same intracellular yeasts (Parente-Rocha et al., 2015). Transcriptional profiling of *Paracoccidioides* yeast showed three different genes annotated as encoding acyl-CoA dehydrogenases, which catalyze the first step in fatty acid breakdown, were increased during respiratory infection of mice (Lacerda Pigosso et al., 2017). One of these was increased 13-fold over yeast grown *in vitro*. However, Parente-Rocha et al. (2015) did not detect any increased acyl-CoA dehydrogenases in *Paracoccidioides* yeasts resident within cultured macrophages. These inconsistencies as well as the potential profiling of a mixed population of intra- and extracellular *Paracoccidioides* yeasts following infection of mice leave the role of fatty acid metabolism unclear for *Paracoccidioides*.

In contrast, *Histoplasma* yeasts within macrophages do not present hallmarks of fatty acid utilization. The transcription of genes encoding enzymes of the glyoxylate cycle (*ICL1* and *MLS1*) as well as the first step in fatty acid β-oxidation [fatty acyl-CoA oxidase (*FOX1)*] were all down-regulated in intracellular *Histoplasma* yeasts (Shen et al., 2020). *Histoplasma* yeasts are unable to metabolize exogenous fatty acids as the carbon source *in vitro*. Thus, changes in β-oxidation enzymes may reflect modulation of endogenous fatty acids unrelated to host carbon substrates. The most conclusive evidence of the lack of host fatty acid consumption by *Histoplasma* yeasts was obtained by functional studies with yeast strains depleted of key enzymes in fatty acid utilization. *Histoplasma* yeasts lacking Icl1 or lacking Fox1 are fully virulent in cultured macrophage infection as well as in respiratory infection of mice (Shen et al., 2020). The lack of any requirement for fatty acid utilization by intracellular *Histoplasma* yeasts differs from that of other fungal pathogens [e.g., *C. albicans* (Lorenz and Fink, 2001) and *C. neoformans* (Kretschmer et al., 2012)], which occupy diverse extracellular niches or are only transiently found within phagocytes. As *Histoplasma* yeasts are nearly all within phagocytes during infection, this suggests that *Histoplasma* yeasts have adapted to a more prolonged residence within the phagosome and do not utilize host fatty acids as a major carbon source, either because exogenous fatty acids are unavailable within the phagosome or because other carbon sources are more consistently obtained in this intracellular compartment. Whether intracellular *Paracoccidioides* yeast metabolism mirrors that of intracellular *Histoplasma* yeasts or if it follows the paradigm of transient occupants of the phagocyte remains to be determined.

### Amino Acid Catabolism

The above studies indicate that both *Paracoccidioides* and *Histoplasma* yeasts exploit gluconeogenic substrates for intracellular proliferation. What are these alternative host carbon substrates obtained from the host macrophage, and specifically which are found within the phagosome? Carbon source utilization tests *in vitro* showed that the spectrum of carbon sources which can be metabolized by yeasts differs from those that are metabolized by *Histoplasma* mycelia, suggesting that the pathogenic yeasts are more metabolically streamlined (Shen et al., 2020). For example, *Histoplasma* yeasts can metabolize hexoses and hexosamines but not pentoses or disaccharides, some of which are metabolized by mycelia. In addition, *Histoplasma* yeasts can use a mix of amino acids as a non-carbohydrate carbon source. Given hexose-catabolism and fatty acids catabolism pathways are not required for *Histoplasma* intracellular proliferation, this focuses attention on amino acids as potential sources of carbon for pathogenic-phase yeasts within the phagosome. Indeed, the phagosome/phagolysosome is a degradative organelle harboring numerous proteases that could generate amino acids and short peptides through host protein degradation.

Expression of genes involved in amino acid biosynthesis hints to the absence of certain amino acids within the phagosome. Bailão et al. (2006) showed that the gene catalyzing the last step of glutamine synthesis (i.e., *GLN1*) in *Paracoccidioides* is up-regulated in fresh human blood, suggesting that glutamine in the human blood environment is not sufficient to support *Paracoccidioides* growth. However, the intracellular residence of *Paracoccidioides* is unknown in this study. In peritoneal macrophages, *Paracoccidioides* yeasts up-regulated the gene involved in methionine synthesis (*METG*) suggesting that methionine is not available within macrophages (Tavares et al., 2007). Differential gene expression of *Paracoccidioides* from respiratory infection showed genes encoding enzymes involved in the metabolism of glycine, serine, threonine, methionine, and lysine are down-regulated (Lacerda Pigosso et al., 2017). Also, down-regulated were genes encoding 4-hydroxyphenylpyruvate dioxygenase (*HPD1*) and homogentisate dioxygenase (*HGD1*), which are involved in phenylalanine and tyrosine degradation suggesting these aromatic amino acids are not consumed from the host. On the other hand, Parente-Rocha's study of *Paracoccidioides* yeasts from macrophages showed increased abundance of Hpd1 (Parente-Rocha et al., 2015) contradicting the *in vivo* gene expression study. Expression studies thus do not provide definitive answers, especially considering the interconversion of many amino acids. Functional evidence comes from a study that used the chemical compound CP1, which is known to inhibit chorismate synthase, the key enzyme in biosynthesis of aromatic amino acids (i.e., tyrosine, tryptophan, and phenylalanine). Treatment of *Paracoccidioides*-infected mice with CP1 reduced fungal burdens ~10-fold compared to untreated mice (Rodrigues-Vendramini et al., 2019) suggesting that *Paracoccidioides* yeasts must synthesize at least one of the three to make up for the deficiency in the environment in which *Paracoccidioides* is found during lung infection.

*Histoplasma* yeasts can catabolize amino acids as the sole carbon source demonstrating the potential to use host amino acids within the phagosome. On the other hand, *Histoplasma* yeasts cannot utilize model host protein substrates for carbon (e.g., bovine serum albumin, gelatin, and hemoglobin). Prior digestion of these proteins with proteinases, including the phagosomal proteinase Cathepsin D, renders the proteins to a state that can support the growth of *Histoplasma* yeasts (Shen et al., 2020). Thus, it appears that *Histoplasma* yeasts can take up amino acids and select peptides to use as carbon but does not metabolize intact proteins. This would be consistent with residence within the phagosome where host proteinases may supply the degradative capacity to liberate amino acids for consumption by intraphagosomal *Histoplasma* yeasts. Indeed, the expression of many metabolic genes of *Histoplasma* yeasts within the macrophage is highly similar to the profile of *Histoplasma* yeasts growing on amino acids *in vitro* (Shen et al., 2020). The expression of various peptidases are increased in *Paracoccidioides* yeast within J774 macrophages (Parente-Rocha et al., 2015) and *Paracoccidioides* yeast secrete a serine proteinase during infection (Lacerda Pigosso et al., 2017), suggesting that proteins and peptides are hydrolyzed to amino acids before consumption. Nonetheless, not all amino acids are sufficiently present in the phagosome to support fungal growth. A *Histoplasma* alanine auxotroph showed impaired virulence in both macrophages and *in vivo*, indicating alanine is deficient in the phagosomal environment (Shen et al., 2020). Combined with the lack of hexose and fatty acid catabolism described for *Histoplasma* yeasts above, it is likely that *Histoplasma* yeasts within the phagosome catabolize amino acids derived from the host which are then processed through gluconeogenic pathways to supply carbohydrates for biomass (Figure 1). Further investigations will be needed to define which amino acids are available within the phagosome and which are catabolized by the intracellular yeasts.

## Intraphagosomal Nitrogen and Sulfur

One of the advantages of amino acid catabolism within the phagosome is that amino acids provide yeasts not only carbon, but also a source of nitrogen and potentially sulfur. Consistent with amino acids providing nitrogen to intracellular yeasts, *Paracoccidioides* yeasts recovered from macrophages have increased abundance of various aminotransferases (Parente-Rocha et al., 2015). However, in *Paracoccidioides* yeasts from lung infections, a branched-chain amino acid aminotransferase is down-regulated again showing inconsistencies between the lung and the macrophage models. The transcriptome of yeasts from lungs showed increased expression of amino acid permeases and peptide transporters (Lacerda Pigosso et al., 2017) consistent with yeasts importing host amino acids and peptides during infection. Differential gene expression analysis of *Histoplasma* yeasts compared to mycelia also show three genes encoding aminotransferases are up-regulated in yeasts (Edwards et al., 2013). Interestingly, studies of *Paracoccidioides* yeasts consistently highlight formamidase as one of the most up-regulated genes or proteins increased in expression during infection of mice or cultured macrophages (Parente-Rocha et al., 2015; Lacerda Pigosso et al., 2017). This suggests formamidase is a central step in nitrogen acquisition and metabolism for *Paracoccidioides* yeast during infection. Alternatively, formamidase may be particularly repressed in the conditions used for *in vitro* growth used as comparison. While the *in vivo* substrate(s) for formamidase is/are unknown (e.g., formamide), arylformamidases are involved in tryptophan degradation which may signal utilization of tryptophan during infection.

Cysteine and methionine represent sulfur-containing amino acids that can provide organic sulfur to fungal pathogens. Changes in cysteine metabolism during transitions between *Histoplasma* yeast and mycelial phases are well-known (Stetler and Boguslawski, 1979; Maresca et al., 1981). Despite a pathway for inorganic sulfur assimilation (e.g., sulfate) that is operative in *Histoplasma* mycelia, *Histoplasma* yeasts require an organic sulfur source which can be supplied by cysteine (Salvin, 1949). Comparison of *Histoplasma* yeast and mycelial transcriptomes showed increased expression of a permease for methionine and cysteine (Hwang et al., 2003), suggesting the pathogenic yeast state is primed to uptake exogenous sulfur-containing amino acids. Despite the auxotrophy of *Histoplasma* yeasts for organic sulfur, *Histoplasma* is fully virulent indicating that organic sulfur is available to *Histoplasma* yeasts within the phagosome. While the ultimate source of organic sulfur for intracellular fungal growth remains unknown, cysteine is a logical candidate, which may be derived from protein degradation or even hydrolysis of abundant glutathione in the macrophage.

## Acquisition of Micronutrients

### Essential Vitamins

While required in smaller quantities than carbon or nitrogen, vitamins are essential micronutrients for growth. This nutritional requirement can be met either by vitamin acquisition from the host or through *de novo* synthesis by fungal cells. Biosynthesis of vitamins by *Paracoccidioides* and *Histoplasma* appears to be the primary source for these essential co-factors. *Paracoccidioides* yeasts infecting the murine lung up-regulate the gene involved in thiamine biosynthesis (*THI13*), suggesting that thiamine from the host is not available to yeasts during infection (Lacerda Pigosso et al., 2017). Parente-Rocha et al. (2015) similarly found that *Paracoccidioides* yeasts within macrophages have increased expression of enzymes involved in the synthesis of thiamine as well as pyridoxine and riboflavin. Although increased expression of these enzymes suggests yeasts synthesize vitamins from simpler metabolic intermediates during infection, expression was measured relative to yeast growth *in vitro* in a vitamin-rich medium. More direct evidence for the lack of vitamins within the macrophage host comes from functional studies in *Histoplasma*. A forward genetics screen for mutants unable to proliferate within macrophages isolated a mutant with a disruption of the *RIB2* gene, which encodes a deaminase catalyzing the third step of riboflavin biosynthesis (Garfoot et al., 2014). While *Histoplasma* yeast lacking Rib2 function could infect macrophages, they were severely attenuated for growth in macrophages as well as attenuated in lung infection. This indicates that *Histoplasma* cannot acquire sufficient exogenous riboflavin from the phagosomal environment to support its intracellular growth. In addition, depletion of the pantothenate biosynthesis enzyme, Pan6, impaired *Histoplasma* virulence in mice indicating that pantothenate is also scarce in the phagosomal environment (Garfoot et al., 2014). On the other hand, depletion of biotin biosynthesis did not affect *Histoplasma* virulence. Although this may suggest that host biotin can be scavenged within the phagosome, it is also possible that the *Histoplasma* yeast had stored sufficient biotin from the pre-growth. In support of this, depletion of biotin synthase (Bio2) did not impair growth *in vitro* until yeasts were passaged multiple times in biotin-deficient media. Thus, the macrophage phagosome compartment in general lacks essential vitamins thereby forcing intracellular yeasts to rely on *de novo* synthesis to meet this nutritional requirement.

### Trace Metals

In addition to vitamin co-factors, trace metals are essential for yeast growth and are variably available within the phagosome depending on the activation state of macrophages. Iron acquisition is essential for *Histoplasma* and *Paracoccidioides* virulence. Attenuation of *Paracoccidioides* and *Histoplasma* yeast growth in macrophages by chloroquine, which impairs phagosome acidification and availability of iron, is reversed by supplementation with exogenous iron (Newman et al., 1994; Dias-Melicio et al., 2006). In addition, supplementation of exogenous iron exacerbated *Paracoccidioides* infection in mice (Parente et al., 2011). Iron restriction of *Paracoccidioides* yeasts *in vitro* induces genes encoding enzymes involved in siderophore biosynthesis and transport (Parente et al., 2011; Silva-Bailão et al., 2014). However, induction of siderophore biosynthesis and transport was not observed in *Paracoccidioides* yeasts isolated from murine macrophages or *in vivo*. Among different iron acquisition mechanisms, *Histoplasma* yeasts secrete siderophores for scavenging rare iron (Hwang et al., 2008; Hilty et al., 2011). Genes encoding siderophore synthesis enzymes are located in a genetic cluster which is activated under low iron conditions through the action of Sre1, a GATA-family transcription factor (Hwang et al., 2008). Loss of siderophore biosynthesis impairs intracellular *Histoplasma* growth indicating the phagosome is a low iron compartment. However, studies of *Histoplasma* infection of mice indicate that siderophores are not required for proliferation of *Histoplasma* pathogenesis until 2-weeks post-infection which coincides with the adaptive immunity stage (Hwang et al., 2008). These results indicate that during early infection, sufficient iron is found within the macrophage phagosome, but after activation of host cells, iron becomes limiting for fungal growth.

Similar to iron, zinc levels in the phagosome can change depending on the activation state of macrophages. Initially, zinc is available for intracellular *Histoplasma* yeast growth, but becomes limiting as macrophages are activated by the GM-CSF cytokine to produce zinc-sequestering metallothioneines (Subramanian Vignesh et al., 2013). *Histoplasma* yeasts produce the Zrt2 zinc transporter to acquire zinc from the environment and this transporter is required for *Histoplasma* virulence in mice, but only after 3 days of infection (Dade et al., 2016). These data indicate that host macrophages can use restriction of trace metals from the phagosome to combat intracellular proliferation of yeasts, particularly after macrophage activation.

**Figure 2:**
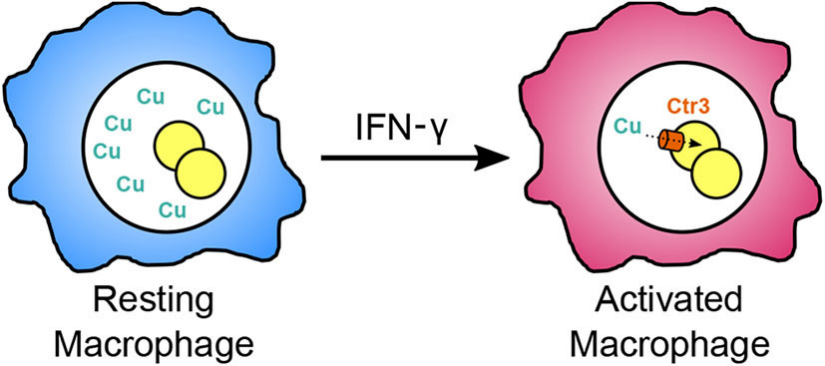
Restriction of available copper within the phagosome following macrophage activation. During initial infection, *Histoplasma* infects resting macrophages (blue), and resides within a phagosomal compartment with sufficiently available copper (green Cu) for fungal proliferation without requiring the high affinity copper transporter Ctr3. Upon activation of macrophages by IFN-γ during adaptive immunity (magenta), copper in the phagosome becomes restricted forcing *Histoplasma* yeast to rely on Ctr3 to import copper.

Host macrophages can use both abundance and restriction of available copper to curtail intracellular pathogen growth. Tavares et al. (2007) found that *Paracoccidioides* yeasts down-regulate a high-affinity copper transporter by 17-fold within cultured macrophages after 6 h, suggesting that the *Paracoccidioides*-containing phagosome has sufficient copper to support *Paracoccidioides* growth. This same down-regulation of the copper transporter was also observed during lung infection of mice at 6 h (Lacerda Pigosso et al., 2017) suggesting the lung environment has ample copper during early infection. The *Histoplasma* genome encodes a homologous copper transporter (Ctr3) whose transcription is induced at low copper concentrations (<150 nM) (Shen et al., 2018). Through use of a transcriptional fusion of the copper-repressible *CTR3* promoter with a fluorescent protein, available copper levels within the *Histoplasma*-containing phagosome could be estimated. In resting macrophages, including alveolar macrophages which *Histoplasma* cells initially encounter during infection, phagosomal copper levels are roughly 300 nM. At this concentration, *Histoplasma* yeasts can acquire sufficient copper without needing the Ctr3 transporter. Upon activation of macrophages with IFN-γ, the available copper in the phagosome significantly decreases resulting in up-regulation of the Ctr3 transporter (Shen et al., 2018). Consistent with the phagosomal copper levels inferred from transcription studies, the Ctr3 transporter is not required for *Histoplasma* proliferation within resting macrophages (i.e., higher phagosomal copper levels) but becomes necessary following macrophage activation as phagosomal copper becomes restricted (Figure 2; Shen et al., 2018). These results indicate that copper availability in the macrophage phagosome, as well as that of iron and zinc, is dynamic and that activation of macrophages imposes a metal restriction strategy to impair the intracellular proliferation of yeasts.

## Conclusion

Initial studies on fungal pathogens often focused on virulence mechanisms for host cell infection and survival. The studies highlighted above indicate that mechanisms of fungal metabolism are an integral component of intracellular fungal pathogenesis. While *in vitro* culture of fungi has typically used glucose- and peptone-rich media, the study of long-term phagosomal residents like *Histoplasma* and *Paracoccidioides* yeasts is revealing that alternate substrates, not hexoses, are available within the phagosome compartment. Furthermore, these dimorphic fungi have tuned their metabolism to exploit these resources during yeast infection of host cells. Consequently, media used for *in vitro* propagation of the yeasts should be re-evaluated for their physiological relevance to the nutritional conditions actually experienced by the yeast cells. The more-specialized metabolism used by yeasts within the phagosome creates opportunities for anti-fungal interventions. The finding that some micronutrient levels in the phagosome can change following macrophage activation and subsequent control of intracellular fungal proliferation further underscores the potential of targeting metabolic pathways for therapeutic control of these primary pathogens.

## Author Contributions

QS and CR conceived of the review topic, performed the literature analysis, and composed the review article. All authors contributed to the article and approved the submitted version.

## Conflict of Interest

The authors declare that the research was conducted in the absence of any commercial or financial relationships that could be construed as a potential conflict of interest.
